# Late Gadolinium Enhancement and Electrocardiographic Associations in Hypertrophic Cardiomyopathy

**DOI:** 10.1111/anec.70077

**Published:** 2025-06-04

**Authors:** Issa Asfour, Shahid Karim, Sair A. Tabraiz, Anwar Chahal, Mohammed Y. Khanji, Akil A. Sherif, Steve R. Ommen, Virend K. Somers, Grace Lin, Peter A. Brady

**Affiliations:** ^1^ East Tennessee State University Johnson City Tennessee USA; ^2^ Mayo Clinic Rochester Minnesota USA; ^3^ William Harvey Research Institute, Barts Biomedical Centre Queen Mary University London London UK; ^4^ University of Pennsylvania Philadelphia Pennsylvania USA; ^5^ Center for Inherited Cardiovascular Diseases WellSpan Health York PA USA; ^6^ Barts Heart Centre, St Bartholomew's Hospital Barts Health NHS Trust London UK; ^7^ Newham University Hospital Barts Health NHS Trust London UK; ^8^ Saint Vincent Hospital Worcester USA; ^9^ Advocate Illinois Masonic Medical Centre Chicago Illinois USA

**Keywords:** atrial fibrillation, cardiac MRI, electrocardiogram, hypertrophic cardiomyopathy, late gadolinium enhancement, T‐wave inversion

## Abstract

**Background:**

Late gadolinium enhancement (LGE) on cardiovascular magnetic resonance (CMR) imaging is a well‐established indicator of myocardial fibrosis in hypertrophic cardiomyopathy (HCM). However, its association with electrocardiographic (ECG) abnormalities and the risk of atrial fibrillation (AF) remains uncertain.

**Objectives:**

To investigate the association between the presence and burden of LGE with ECG characteristics, including precordial voltage, depolarization and repolarization abnormalities, and the incidence of AF in adults with HCM.

**Methods:**

We conducted a retrospective cohort study of 144 adults with HCM with CMR and 12‐lead ECG within 30 days of each other. LGE was quantified as a percentage of LV mass and categorized as absent, < 5%, or ≥ 5%. ECG parameters, including QRS voltage, repolarization abnormalities, and LVH criteria, were analyzed. Incident AF was assessed during a median follow‐up of 6.6 years.

**Results:**

LGE was present in 96 (67%) patients, with 21 (22%) having ≥ 5% LGE. There were no significant differences in precordial voltage between patients with and without LGE across Sokolow‐Lyon, Cornell, and Romhilt‐Estes criteria. However, T‐wave inversion was more common in leads I (41% vs. 19%, *p* = 0.009), aVL (50% vs. 31%, *p* = 0.033), and V4 (41% vs. 23%, *p* = 0.035) in patients with LGE. Patients with ≥ 5% LGE had a significantly lower median LVEF (64% vs. 74%, *p* = 0.003). Additionally, LGE presence was not associated with an increased risk of incident AF (HR 1.8, 95% CI 0.6–5.3, *p* = 0.308).

**Conclusion:**

In contrast to pediatric HCM, LGE is associated with specific ECG repolarization abnormalities, particularly T‐wave inversion in lateral leads, but does not significantly affect precordial voltage in adults.

## Graphical abstract

Late gadolinium enhancement (LGE) is a negative prognostic marker in hypertrophic cardiomyopathy (HCM). In pediatric patients with HCM, greater LGE presence is associated with a paradoxical reduction in left ventricular precordial lead voltages on the electrocardiogram. This longitudinal study of 144 adult patients with HCM found no such relationship between LGE presence and pseudo‐normalization of LV precordial voltages nor the incidence nor prevalence of atrial fibrillation, but identified T‐wave inversion, particularly in leads I, aVL and V4, was most predictive of LGE presence.

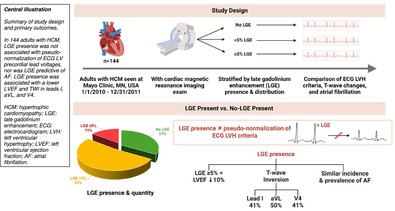

**Abbreviations**
AF: atrial fibrillation
AHA: American Heart Association
BP: blood pressure
CMR: cardiac magnetic resonance
CRT: cardiac resynchronization therapy
ECG: electrocardiogram
EDV: end diastolic volume
EDVI: end diastolic volume index
HCM: hypertrophic cardiomyopathy
ICD: implantable cardioverter defibrillator
LGE: late gadolinium enhancement
LV: left ventricle
LVEF: left ventricular ejection fraction
MI: myocardial infarction
NYHA: New York Heart Association
PPM: permanent pacemaker
SCD: sudden cardiac death

## 1 Introduction

Hypertrophic cardiomyopathy (HCM), the most common inherited cardiomyopathy with an estimated population prevalence of 1 in 500 (Liew et al. 2017), is clinically characterized by left ventricular hypertrophy not explained by loading conditions. Cardiovascular magnetic resonance (CMR) imaging is considered the gold standard diagnostic technique to study left ventricular (LV) volume and mass as it provides vivid and comprehensive visualization of LV hypertrophy (Grothues et al. 2002). CMR can also be used to detect myocardial fibrosis with the use of late gadolinium enhancement (LGE) throughout the heart, including the papillary muscles (Moon et al. 2004). Approximately 75%–95% of patients with HCM will have an abnormal 12‐lead electrocardiogram (ECG) (Maron 2002).

There remains, however, an important knowledge gap as to how the presence, distribution, and quantity of LGE (and therefore fibrosis in the case of HCM) (Aquaro et al. 2023) may affect the 12‐lead ECG in the adult population, and whether LGE can act as a variable predictive of future atrial fibrillation (AF) events. A study of pediatric patients (*n* = 37) with HCM reported pseudo‐normalization of LV precordial lead voltages when LGE was present despite greater septal thickness (Guerrier et al. 2016). Thus, we sought to investigate the relationship between the presence of LGE and LV precordial voltage changes, depolarization and repolarization abnormalities, and to determine the relationship between LGE presence and AF in adults with HCM.

## 2 Methods

### 2.1 Study Population

This retrospective cohort study was approved by the institutional review board of Mayo Clinic. We identified all consecutive patients with a diagnosis of HCM, seen at Mayo Clinic, Rochester, Minnesota over a 2‐year period (January 1, 2010, to December 31, 2011), including new cases and those undergoing follow‐up (*n* = 176). Diagnostic criteria included left ventricular wall thickness ≥ 15 mm when assessed by echocardiography or cardiac MRI in the absence of other systemic or cardiac causes. The following inclusion criteria had to be met: ≥ 18 years old, with a CMR study and 12‐lead ECG performed within 30 days of each other. Patients with a history of cardiac surgery, aortic stenosis, or sub‐aortic membrane were excluded. Potential ECG confounders such as prior myocardial infarction (MI), treatment with digoxin, left bundle branch block, complete right bundle branch block, QRS duration > 120 ms, paced rhythm, and BMI < 18 or > 35 kg/m^2^ were also excluded.

### 2.2 Data Variables and Abstraction

Demographic, clinical, and outcome data were abstracted through exhaustive review of electronic healthcare records. ECGs were reviewed in a blinded fashion for baseline data including ventricular rate, QRS axis (degrees), QRS duration (ms), and QT interval (ms). ECGs were also analyzed for LV precordial voltage using the Sokolow‐Lyon (Sokolow and Lyon 1949), Cornell (Truong et al. 2010), and Romhilt‐Estes (Romhilt and Estes Jr. 1968) criteria. Additionally, non‐voltage characteristics including pathological Q wave, T‐wave inversion, and ST segment depression were recorded.

### 2.3 CMR Acquisition

All studies were performed on a 1.5 Tesla CMR scanner (Signa Twin Speed Excite, General Electric, Waukesha, Wisconsin). CMR data were analyzed by experienced reporters without knowledge of clinical parameters. LV ejection fraction (LVEF), LV stroke volume and end‐diastolic volumes, LV mass, and maximal (end‐diastolic) septal thickness were traced and recorded from short‐axis views (8‐mm slice thickness, 1‐mm gap) of standard ECG‐gated steady‐state free precession pulse sequence with the following characteristics: repetition time = 3.5 ms, echo time = 1.6 ms, temporal resolution = 40 ms, matrix 224 × 160, flip angle = 45°, bandwidth = 125 Hz, views per segment = 8 to 16.

Images for detection of hyper‐enhancement were obtained around 10 min after injecting double‐dose intravenous gadodiamide (0.2 mmol/kg) using a segmented inversion recovery prepared fast gradient echo sequence. The prescription for this sequence was identical to the short‐axis cine sequence to ensure optimal image resolution. Scan parameters were as follows: repetition time = 6.5 ms, echo time = 3.1 ms, inversion time = 160 to 240 ms, matrix = 256 × 192, 8 mm section thickness with 1 mm interslice gap, field of view 33–44 cm, flip angle = 20°, bandwidth = 31.2 kHz, views per segment = 24. A cine multi‐inversion time inversion recovery sequence (Cine IR) was used to select the optimal inversion time for LGE imaging. Each study was re‐read with chamber volumes calculated and quantification of LGE calculated by segment using previously reported methods, whereby an MR gray‐scale threshold of ≥ 6 standard deviations above the mean signal intensity for normal myocardium was interpreted as LGE in Circle CVI 42 (Circle Cardiovascular Imaging Inc., Calgary, AB, Canada), with readers masked from ECG findings. Figures S1 and S2 provide examples of patients' CMR exams and ECGs that were studied (Harrigan et al. 2011).

The extent of LGE was defined as the percentage of LV mass affected by gadolinium enhancement. While ≥ 15% LGE extent has historically been considered the prognostic threshold beyond which there is a high risk of SCD, more recent studies have suggested other factors such as dispersion or irregularity of the shape of myocardial fibrosis may also confer additional risk. Importantly, a number of studies have also since reported ≥ 5% LGE extent to be associated with a significantly higher likelihood of arrhythmic cardiac events including SCD compared to those with < 5% LGE, by a factor of seven‐fold (hazard ratio for SCD [95% CI], 7.1 [2.7, 18.8], *p* < 0.001) (Greulich et al. 2021). We therefore allocated patients to one of three groups for comparison: no LGE, < 5% LGE, and ≥ 5% LGE (Wang et al. 2023).

### 2.4 Follow‐Up

Outcome data was collected until December 31, 2019. New incidences of AF (ECG or monitor recorded sustained AF > 30 s) (Kirchhof et al. 2016), and device interrogation data were also collected. Other data recorded in an exploratory manner included the development of sustained (VT), non‐sustained ventricular tachycardia (NSVT), or sudden cardiac death (SCD) during the follow‐up period, noting the study was likely underpowered to assess the incidence of these events based on LGE presence. Median follow‐up duration was 6.6 years (IQR: 1.7, 9.9 years).

### 2.5 Statistical Analysis

Continuous variables are presented as mean and standard deviation or median and quartiles, based on distribution. Categorical variables are presented using frequency and percentage. Inter‐group comparisons of continuous variables were conducted using the two‐sample *t*‐test or analysis of variance (ANOVA) where normally distributed, and Wilcoxon rank‐sum or Kruskal‐Wallis test if skewed, while Pearson's *χ* ^2^ tests were used for categorical variables. Patients were further subdivided according to the extent of LGE. Cox regression analysis using the log‐rank test was used to compare the risk of incident AF between groups. SAS Version 9.4 was used for all analyses and *p*‐values < 0.05 were considered to be statistically significant.

## 3 Results

In total, 176 patients underwent CMR examination as part of their evaluation with an ECG taken within 30 days either side of the CMR scan. Of these, 26 patients were excluded as an alternative cause for their hypertrophy was diagnosed, and 6 were excluded as their CMR examinations were not of sufficient quality to allow LGE classification. One hundred and forty‐four patients were therefore included in the final study sample. The mean age of subjects was 55.5 years, and 63% were males. There were no significant between‐group differences in baseline clinical parameters such as age, sex, BMI, history of hypertension, diabetes mellitus, smoking, New York Heart Association (NYHA) class, or family history of HCM or sudden death between patients with or without LGE. Baseline characteristics are summarized in Table 1.

**TABLE 1:** Patient Characteristics.

Variable	Overall *N* = 144	No LGE *N* = 48	LGE < 5% *N* = 75	LGE ≥ 5% *N* = 21	*p*
Age (years)	55.5 (15.4)	57.6 (12.0)	53.7 (17.2)	57.2 (15.6)	0.340
BMI (kg/m^2^)	30.3 (5.1)	30.3 (4.8)	30.7 (5.4)	28.9 (4.1)	0.401
Male	91 (63%)	29 (60%)	50 (67%)	12 (57%)	0.642
Hypertension	61 (42%)	23 (48%)	30 (40%)	8 (38%)	0.771
Diabetes	12 (8%)	4 (8%)	7 (9%)	1 (5%)	0.591
Current smoker	13 (9%)	5 (11%)	7 (10%)	1 (5%)	0.890
Family history of	—	—	—	—	—
HCM	39 (27%)	10 (21%)	23 (31%)	6 (29%)	0.310
Sudden death	34 (24%)	10 (21%)	18 (24%)	6 (29%)	0.181
Genetic testing completed	6 (4%)	0 (0%)	5 (7%)	1 (5%)	0.314
Syncope	20 (14%)	7 (15%)	10 (13%)	3 (15%)	0.125
Chest pain	28 (20%)	10 (21%)	16 (22%)	2 (10%)	0.081
NYHA Class	—	—	—	—	—
Class I	3 (3%)	0 (0%)	2 (5%)	1 (7%)	
Class II	21 (24%)	9 (29%)	11 (25%)	2 (14%)	
Class III	27 (31%)	9 (32%)	12 (27%)	6 (43%)	0.860
Class IV	2 (2%)	1 (4%)	1 (2%)	0 (0%)	
Beta Blocker	97 (67%)	30 (63%)	51 (68%)	16 (76%)	0.532
Calcium Channel Blocker	27 (19%)	13 (27%)	11 (15%)	3 (14%)	0.191
NSVT	27 (19%)	4 (9%)	16 (22%)	7 (33%)	0.091
Prior AF history	10 (7%)	4 (8%)	3 (4%)	3 (14%)	0.201
Incident AF	20/134 (15%)	4/44 (9%)	12/72 (17%)	4/18 (22%)	0.323
Total AF Prevalence	30 (21%)	8 (17%)	15 (20%)	7 (33%)	0.291

*Note:* Values presented are mean (SD) or frequency (percentage).

Abbreviations: AF, atrial fibrillation; BMI, body mass index; HCM, hypertrophic cardiomyopathy; LGE, Late gadolinium enhancement; NSVT, Non‐sustained ventricular tachycardia; NYHA, New York Heart Association.

### 3.1 CMR Findings

CMR findings are summarized in Table 2; 48 (33%) patients had no LGE. Of the 96 patients with LGE present, 75 (78%) patients had < 5%, and 21 (22%) patients had ≥ 5% LGE burden. Median LVEF was 74% and lower in those with LGE ≥ 5% by 10% (median 64%, *p* = 0.003). Distribution of LGE varied among the 96 patients, with the highest quantities distributed in the mid‐inferoseptal (*n* = 86; 90%), mid‐anteroseptal (*n* = 86; 90%) and apical septal segments (*n* = 84; 88%). The lowest quantities of LGE distribution were found in the true apex (*n* = 15; 16%) and mid‐inferolateral segment (*n* = 53; 55%). LGE distribution by specific myocardial segment is reported in Table S1.

**TABLE 2:** Cardiac MRI Findings.

Variable	Overall *N* = 144	No LGE *N* = 48	LGE < 5% *N* = 75	LGE ≥ 5% *N* = 21	*p*
LVEF (%)	74 (67, 78)	75 (68, 78)	74.5 (71, 79)	64 (57, 76)	0.003
LGE (%)	2 (1, 4)	N/A	2 (1, 3)	7 (6, 11)	< 0.001
LVEDV (indexed, ml/m^2^)	63 (52, 73)	60 (51, 70)	63 (53, 72)	64 (53, 82)	0.370
LV stroke volume (indexed, ml/m^2^)	45 (38, 54)	44 (37, 51)	46 (39, 54)	45 (32, 53)	0.370
Maximal wall thickness (mm)	20 (17, 23)	18 (16, 20)	22 (18, 25)	20 (17, 24)	< 0.001
HCM morphology	—	—	—	—	—
Asymmetric	118 (83%)	42 (88%)	57 (78%)	19 (90%)	
Free wall	1 (1%)	0 (0%)	1 (1%)	0 (0%)	
Concentric LVH	5 (4%)	1 (2%)	3 (4%)	1 (5%)	0.690
Apical HCM	18 (13%)	5 (10%)	12 (16%)	1 (5%)	

*Note:* Values presented are median (Q1, Q3), and frequency (percentage).

Abbreviations: HCM, hypertrophic cardiomyopathy; LGE, late gadolinium enhancement; LV, left ventricle; LVEDV, left ventricular end‐diastolic volume; LVEF, left ventricular ejection fraction; LVH, left ventricular hypertrophy.

### 3.2 ECG Findings

ECG characteristics are summarized in Table 3. Pathological Q waves were present in 27 (19%) patients with a median depth of 2.5 mm. One hundred and sixteen (82%) patients had T‐wave inversion, and 28 (20%) patients had ST‐segment depression. Regarding LVH criteria, 73 (52%) patients met Sokolow‐Lyon criteria, 48 (34%) patients met Cornell criteria, and 54 (38%) patients met definite LVH Romhilt‐Estes criteria.

**TABLE 3:** ECG Findings.

Variable	Overall *N* = 144	No LGE *N* = 48	LGE < 5% *N* = 75	LGE ≥ 5% *N* = 21	*p*
Max aVL QRS voltage (mm)	7 (4, 10)	7 (4, 10)	7 (4, 10)	6 (3, 13)	0.770
QRS axis (deg)	20 (−4, 48)	15 (−3, 27)	28 (4, 55)	10 (−24, 60)	0.211
PR (ms)	168 (150, 188)	167 (150, 178)	170 (150, 192)	179 (154, 202)	0.291
QRS duration (ms)	99 (90, 108)	98 (90, 108)	100 (90, 108)	100 (88, 114)	0.593
QTc (ms)	433 (404, 458)	420 (400, 454)	434 (404, 460)	440 (404, 460)	0.331
Q wave (nominal)	27 (19%)	7 (15%)	17 (23%)	3 (14%)	0.454
Q wave (mm)	3 (2, 3)	2 (1, 3)	3 (2, 3)	4 (2, 7)	0.132
T‐wave inversion (nominal)	116 (82%)	35 (74%)	67 (91%)	4 (19%)	**0.013**
ST segment depression	28 (20%)	7 (15%)	17 (23%)	4 (19%)	0.550
LVH criteria	—	—	—	—	—
Sokolow	73 (52%)	20 (43%)	44 (60%)	9 (43%)	0.110
Cornell	48 (34%)	13 (28%)	24 (32%)	11 (52%)	0.131
Romhilt‐Estes	—	—	—	—	0.431
≥ 4 (probable LVH)	8 (6%)	2 (4%)	4 (5%)	2 (10%)	
≥ 5 (definite LVH)	54 (38%)	14 (30%)	33 (45%)	7 (33%)	

*Note:* Values presented are median (Q1, Q3), and frequency (percentage).

Abbreviations: LGE, late gadolinium enhancement; LVH, left ventricular hypertrophy.

There were no significant differences in LV precordial voltage between those with and without LGE in any of the three LVH criteria: Sokolow‐Lyon (*p* = 0.11), Cornell (*p* = 0.13), or Romhilt‐Estes (*p* = 0.43). There were no significant differences in QRS axis (degrees, *p* = 0.21), QRS duration (ms, *p* = 0.59), or QT interval (ms, *p* = 0.33) between patients with LGE versus those without. The presence of LGE was not associated with repolarization disturbances such as ST‐segment depression (*p* = 0.55) nor pathological Q waves (*p* = 0.45). Compared to patients without LGE, those with LGE present were more likely to have T‐wave inversion in leads I (41% vs. 19%, *p* = 0.009), aVL (50% vs. 31%, *p* = 0.033) and V4 (41% vs. 23%, *p* = 0.035). T‐wave inversion based on LGE distribution is reported in Table S2.

### 3.3 Atrial Fibrillation and Late Gadolinium Enhancement

**FIGURE 1:**
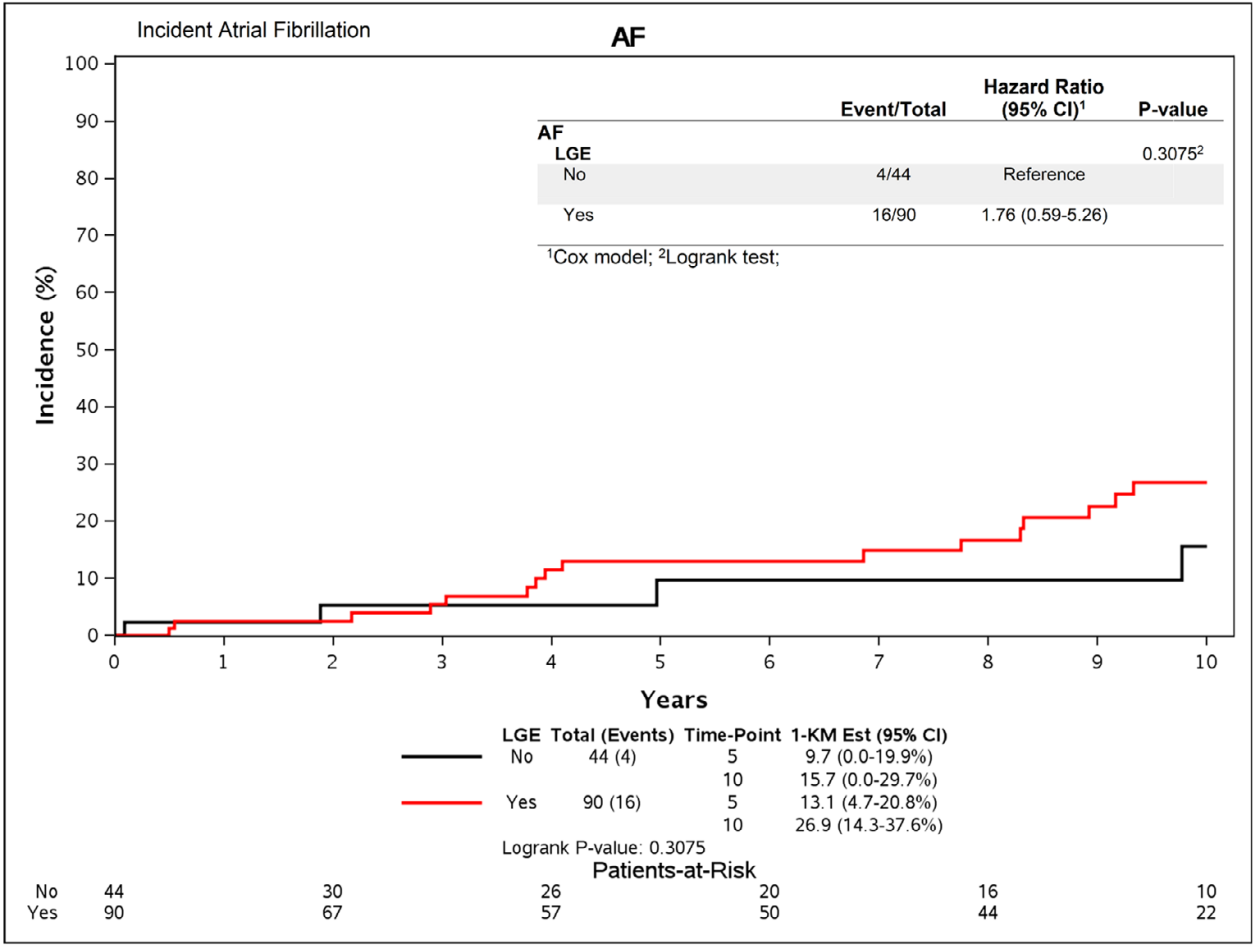
Incident Atrial Fibrillation. Kaplan–Meier plot and log‐rank test comparing cumulative incidence of AF between patients with and without LGE.

Participant medical records were followed up for the incidence of SCD, VT, NSVT, and AF with a median follow‐up period of 6.6 years. There were no confirmed occurrences of SCD, VT, nor NSVT in patients with or without LGE present. Any patients with an established history of AF at the time of imaging (*n* = 10) were censored from this analysis. From the remaining 134 participants, 20 (15%) developed incident AF during the follow‐up period, of whom 4 (9%) had no LGE, 12 (17%) had < 5% LGE, and 4 (22%) had ≥ 10% LGE, *p* = 0.320. The hazard ratio (95% CI) for incident AF during the follow‐up period for patients with LGE present compared to those without LGE was 1.8 (0.6, 5.3, *p* = 0.308) (illustrated in Figure 1). The prevalence of AF was 17% in those with no LGE, 20% in those with < 5% LGE, and 33% in those with ≥ 5% LGE, *p* = 0.288.

## 4 Discussion

A number of important findings arise from this study. First, contrary to the paradoxical phenomenon reported in pediatric patients, LGE presence was not associated with pseudo‐normalization of LV precordial lead voltages in adult patients with HCM. Second, we report repolarization abnormalities (TWI in leads I, aVL, and V4) associated with the presence of LGE. Lastly, we found no association between LGE presence and the incidence or prevalence of AF.

### 4.1 Voltage Criteria of Surface ECG in Myocardial Fibrosis

Although we report an association between maximal LV wall thickness and LGE presence, we found no association between LGE presence and pseudo‐normalization of LV precordial lead voltage in this adult population. Comparing results from this study to a similar study carried out in a pediatric population (ages 9–21 years, *n* = 37 patients). Both our study and Guerrier et al. (2016) reported an association between the presence of LGE and increased septal thickness; however, in the pediatric population, an inverse relationship was described between LGE extent and precordial lead voltages in *S*_V1_ (*p* = 0.005), *R*_V6_ (*p* = 0.005) and *S*_V1_ + *R*_V6_ (*p* = 0.002). The authors attributed their finding of unexpectedly low LVH voltage to the presence of fibrosis. In our study, however, the relationship between LGE presence and precordial voltage was not significant. This difference in findings could be attributed to a number of reasons such as differences in the shape of the thorax or the presence of comparatively more extra‐thoracic fat and muscle in adults rather than children. The ECG may be more sensitive to the presence of fibrosis in the pediatric population. Alternatively, noting the aforementioned study had a relatively small sample size, the findings may feasibly represent a type‐II error.

### 4.2 T‐Wave Inversion in Patients With LGE

Patients with LGE present were more likely to have TWI in leads I, aVL, and V4, with a prevalence of 41% (*p* = 0.009), 50% (*p* = 0.033) and 41% (*p* = 0.035) respectively. Prior studies investigating the relationship between T‐wave inversion and LGE presence in HCM have reported conflicting results. One study comparing LGE presence and TWI in HCM reported a much higher prevalence of TWI in patients with apical hypertrophy morphology when compared to concentric or asymmetrical septal hypertrophy (Song et al. 2013). Another reported an association between TWI, increased apical septal thickness, and increased septum‐to‐free‐wall thickness ratio, but not between apical LGE presence and TWI (Chen et al. 2014).

These conflicting reports suggest other factors may contribute to the development of TWI. Nevertheless, our data suggest the presence of TWI in lateral leads I and aVL may represent an “eyeball” indicator of subsequent fibrosis. CMR is not widely available in all institutions, may be prohibitively costly, and requires technical expertise—patients often have to travel to obtain the study which can be met with reluctance. Using the 12‐lead ECG to identify those at risk of fibrosis may present an additional screening tool and motivator for patients to proceed with CMR, although this would require a prospective validation study.

### 4.3 Prevalence of AF in Patients With Large LGE Burden

We report an overall prevalence of AF of 21% in our study sample. In a prior study of 480 patients with HCM followed for 9.1 ± 6.4 years, 22% of patients developed AF independent of advancing age, congestive symptoms, and LA dimensions at the time of diagnosis. Patients with HCM had a 4‐ to 6‐fold increased likelihood of developing AF compared with the general population (Olivotto et al. 2001). Investigators rationalized this finding by implicating the effect of fibrosis of the LV on its compliance, resulting in an increase in LV filling pressure and subsequently increased LA pressure, promoting structural remodeling, a substrate for AF (Suksaranjit et al. 2015). Similarly, another study reported specific HCM mutations were associated with a higher incidence of AF when compared with genotype‐negative patients and related this to the intrinsic ventricular myopathy that might extend to the atria in association with the mutation (Gruver et al. 1999). In our study, only 6 patients out of 144 had undergone genetic testing (the other patients had declined testing, mostly due to high out‐of‐pocket expenses); of these 6, two patients with LGE presence had mutations in the *MYBPC3* gene, and one patient had a mutation in the *TMP1* gene. The benefits of *prophylactic* anticoagulation initiation in preventing complications of AF in patients with HCM may warrant further investigation given the significantly higher incidence suggested.

### 4.4 Incidence of AF in Patients With LGE

Despite a median follow‐up period of 6.6 years, the association between LGE burden and incidence of AF did not reach significance, likely due to being underpowered. This finding is indirectly supported by multiple prior studies, but none to our knowledge investigated LGE as a prognostic marker for AF. Previously published studies describe the pathogenesis of AF as an interaction between rapidly firing ectopic foci and abnormal atrial tissue substrate capable of maintaining the arrhythmia. AF exerts varying effects on the heart with time, influencing atrial remodeling in multiple ways independently of fibrosis, such as through electrical remodeling through altered expression of cardiac ion channels, and the formation of functional reentry substrate, and may explain the progression of paroxysmal to persistent AF, as well as the concept of an atrial myopathy (Iwasaki et al. 2011).

### 4.5 Reduced EF in Patients With Large LGE Burden

Our study found LVEF was on average 10% lower in those with LGE ≥ 5% (*p* = 0.003), which is in keeping with another study that found patients with LVEF < 55% were 1.3 times more likely to have LGE present (Neubauer et al. 2019). The results of our study support the notion that LGE correlates with regions of fibrosis and that fibrosis is a key step in the development of cardiac dysfunction (Tandon et al. 2015).

### 4.6 Strengths and Weaknesses

Strengths of this study include a 2‐year “real world” longitudinal sample with a lengthy follow‐up period, consistent CMR imaging protocols and LGE quantification methods. We had a relatively large sample size of patients, and all studies were re‐reported by two operators with every 10th study checked by a third operator with minimal inter and intra‐reporter variability. The results of our study should be interpreted in the light of certain limitations. The retrospective nature of this study may introduce selection bias, and may be limited by the completeness of data sources, potentially explaining the lack of confirmed occurrences of NSVT, VT, or SCD, as well as the study likely being underpowered to explore the relationship between LGE presence and these outcomes. Additionally, since all patients were selected from a tertiary‐care center, this may have introduced referral bias. Very few (*n* = 6) patients had undergone genetic testing due to high out of pocket expenses.

While we report maximal wall thickness, the pattern of LV hypertrophy in each group, and the segmental location of LGE in each group, whether each segment affected by LGE was beyond the limits of normal thickness was not assessed. Lastly, we used ≥ 5% LGE to categorize high LGE presence rather than the 15% LGE burden historically recognized to be predictive of SCD risk; however, an LGE burden > 5% of LV mass is itself associated with a significantly elevated risk of SCD in HCM when compared to those with no or < 5% LGE burden, lending credibility to stratification at this level (Greulich et al. 2021).

## 5 Conclusions

Despite substantial advances in imaging, the need for non‐invasive surrogate markers of disease progression persists. In this sample of adult patients with HCM, LGE presence was associated with increased septal thickness and reduced LVEF. However, LGE presence was not associated with a reduction in LV precordial lead voltages nor an increased likelihood of AF. The presence or development of T‐wave inversion in leads I, aVL, and V4 was indicative of LGE and may serve as useful indicators to prompt further investigation and risk assessment.

## Author Contributions

The author takes full responsibility for this article.

## Conflicts of Interest

The authors declare no conflicts of interest.

## Supporting information


**Appendix S1.** **Figure S1a,b.** Exemplars of analyzed ECGs and CMRs.
**Figure S2a,b.** Patient 3 is a 47‐year‐old male diagnosed with HCM at age 31 following extensive family history (3 uncles and 4 of 6 siblings diagnosed with HCM).
**Table S1.** Segmental distribution of late gadolinium enhancement.
**Table S2.** ECG T‐wave inversion prevalence, based on late gadolinium enhancement.

## Data Availability

The data underlying this article will be shared upon reasonable request to the corresponding author.
