# Supplementary material for: Late Gadolinium Enhancement and Electrocardiographic Associations in Hypertrophic Cardiomyopathy
Source: Ann Noninvasive Electrocardiol. 2025 Jun 4;30(4):e70077. doi: 10.1111/anec.70077 (PMC12134768; doi:10.1111/anec.70077)
Supplement: Supplementary file 1 — Appendix S1. Figure S1a,b. Exemplars of analyzed ECGs and CMRs. Figure S2a,b. Patient 3 is a 47‐year‐old male diagnosed with HCM at age 31 following extensive family history (3 uncles and 4 of 6 siblings diagnosed with HCM). Table S1. Segmental distribution of late gadolinium enhancement. Table S2. ECG T‐wave inversion prevalence, based on late gadolinium enhancement. [file ANEC-30-e70077-s001.pdf]

Late Gadolinium Enhancement and Electrocardiographic Associations in Hypertrophic Cardiomyopathy

-Supplemental Tables and Figures-

Supplemental Table 1. LGE Segmental Distribution

Myocardial Segment	Overall N=144	No LGE N=48	LGE<5% N=75	LGE≥5% N=21	P Value
Basal segments					
Anterior	62 (65%)	--	44 (59%)	18 (86%)	**0.002**
Anteroseptal	77 (80%)	--	57 (76%)	20 (95%)	**0.050**
Inferoseptal	78 (81%)	--	58 (77%)	20 (95%)	0.061
Inferior	70 (73%)	--	50 (67%)	20 (95%)	**0.009**
Inferolateral	60 (63%)	--	42 (56%)	18 (86%)	**0.013**
Anterolateral	54 (56%)	--	39 (52%)	15 (71%)	0.110
Mid Segments					
Anterior	63 (66%)	--	48 (64%)	15 (71%)	0.531
Anteroseptal	86 (90%)	--	66 (88%)	20 (95%)	0.343
Inferoseptal	86 (90%)	--	66 (88%)	20 (95%)	0.343
Inferior	82 (85%)	--	63 (84%)	19 (90%)	0.463
Inferolateral	53 (55%)	--	39 (52%)	14 (67%)	0.230
Anterolateral	57 (59%)	--	43 (57%)	14 (67%)	0.441
Apical Segments					
Anterior	68 (71%)	--	50 (67%)	18 (86%)	0.090
Septal	84 (88%)	--	64 (85%)	20 (95%)	0.231
Inferior	76 (79%)	--	56 (75%)	20 (95%)	**0.043**
Lateral	56 (58%)	--	43 (57%)	13 (62%)	0.710
Apex	15 (16%)	--	11 (15%)	4 (19%)	0.633

Values are presented as frequency (percentage). LGE: late gadolinium enhancement; AHA: American Heart Association.

Supplemental Table 2. T-Wave Inversion and LGE Distribution

ECG Lead	No LGE N=48	LGE present N=96	P Value
Lead I	9 (19%)	39 (41%)	**0.009**
Lead II	10 (21%)	27 (28%)	0.351
Lead III	10 (21%)	21 (22%)	0.892
Lead aVR	14 (29%)	30 (31%)	0.800
Lead aVL	15 (31%)	48 (50%)	**0.033**
Lead aVF	8 (17%)	8 (8%)	0.131
Lead V1	14 (29%)	17 (18%)	0.113
Lead V2	6 (13%)	19 (20%)	0.284
Lead V3	8 (17%)	20 (21%)	0.550
Lead V4	11 (23%)	39 (41%)	**0.035**
Lead V5	14 (29%)	42 (44%)	0.091
Lead V6	11 (23%)	29 (30%)	0.362

Values are presented as frequency (percentage). LGE: Late gadolinium enhancement.

Supplemental Figures 1 & 2. Exemplars of analyzed ECGs and CMRs.

Supplemental Figure 1

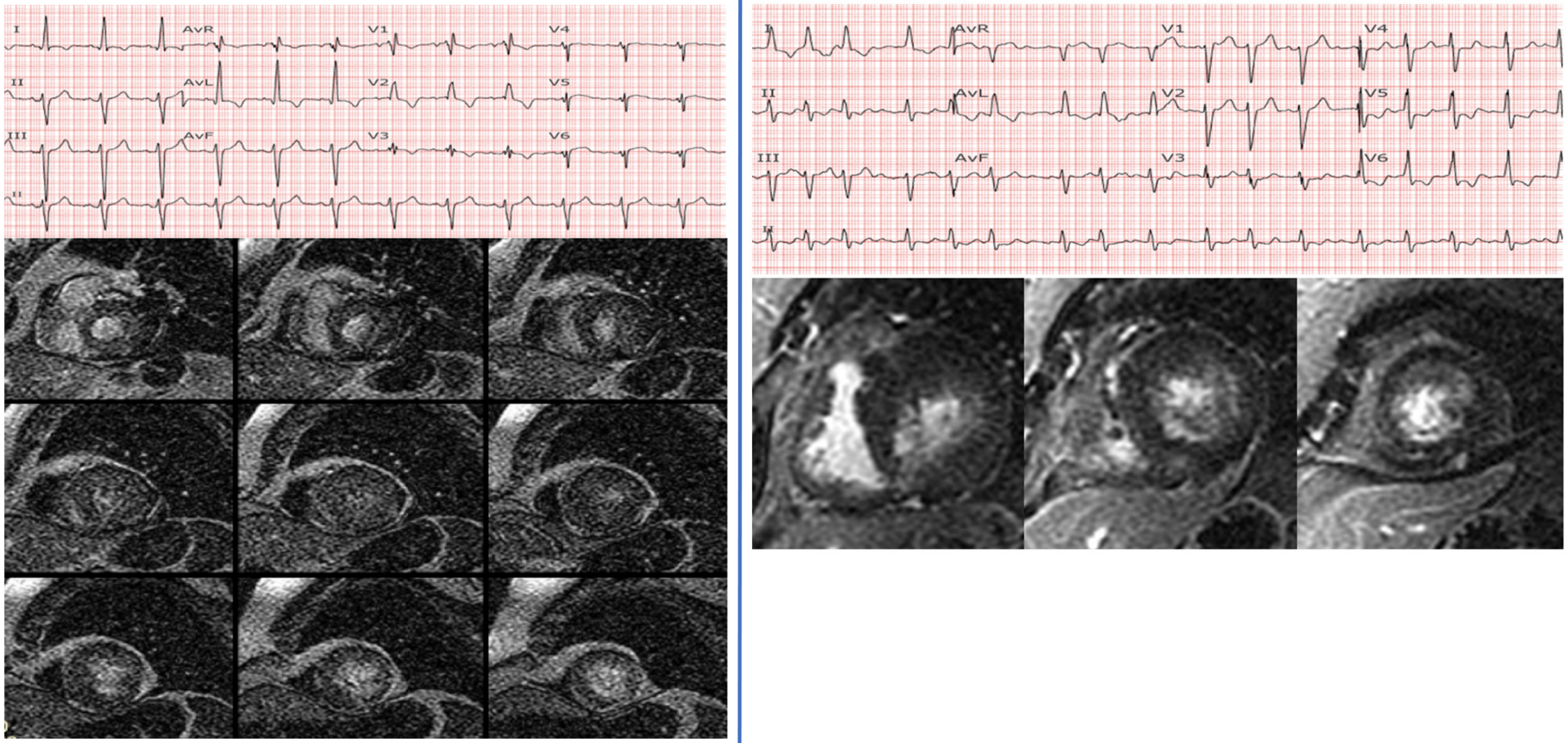

a. Patient 1

b. Patient 2

1a. *Patient 1 is a 54-year-old male recently diagnosed with HCM with a history of sleep apnea, obesity, and hypertension, found to have a 90mmHg mid-cavitary gradient with mid-cavitary obliteration during systole. Short axis CMR images are demonstrated.* ***1b.*** *Patient 2 is a 51-year-old female with mutation in exon 16 of the Myosin Heavy Chain gene (MYH7). Short axis CMR images demonstrate LGE in basal, mid, and apical LV segments (from left to right respectively).*

Supplemental Figure 2

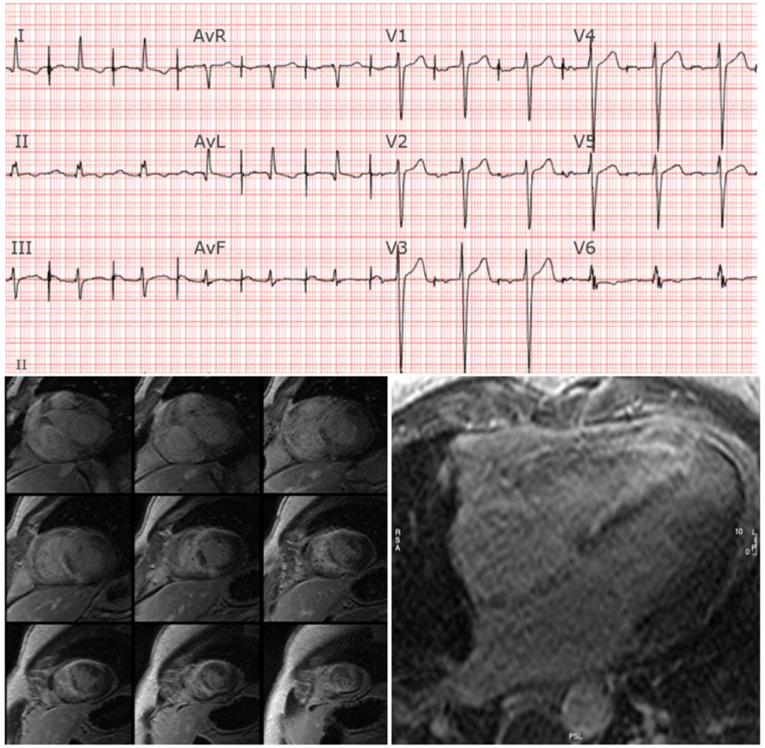

a. Patient 3

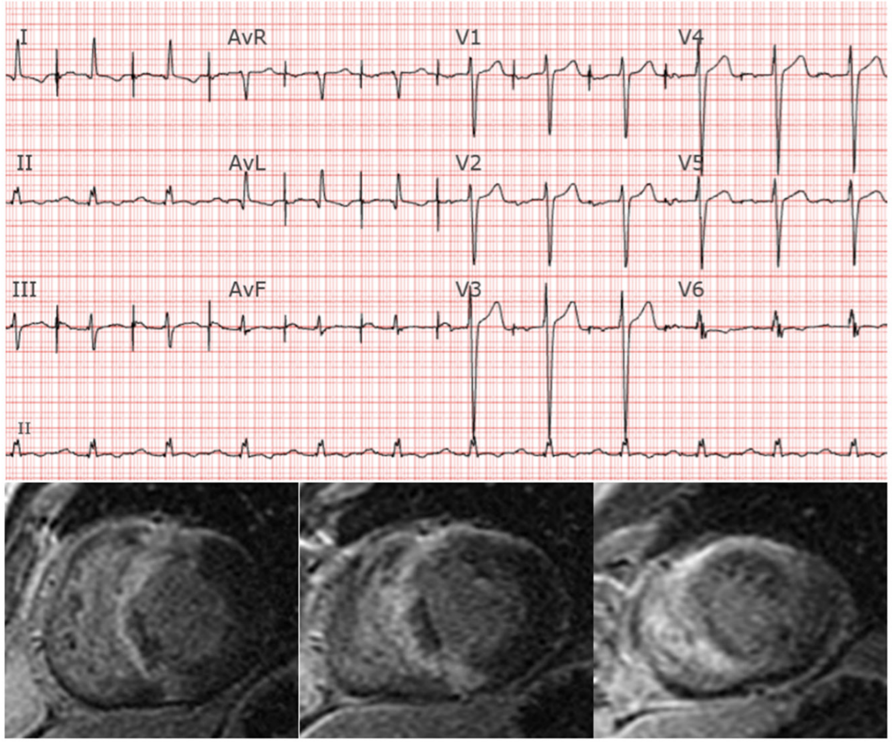

b. Patient 3

Figure 2a-b. *Patient 3 is a 47-year-old male diagnosed with HCM at age 31 following extensive family history (3 uncles and 4 of 6 siblings diagnosed with HCM). The patient also suffered from a cardiac arrest with subsequent ICD placement and atrial fibrillation. Alpha Tropomyosin (ASP175ASN) genetic mutation was identified. The CMR images demonstrate extensive late gadolinium enhancement (LGE) increasing from left to right, with extensive septal involvement.*
